# H3K4me3 mediates uterine leiomyoma pathogenesis via neuronal processes, synapsis components, proliferation, and Wnt/β-catenin and TGF-β pathways

**DOI:** 10.1186/s12958-023-01060-2

**Published:** 2023-01-26

**Authors:** María Cristina Carbajo-García, Elena Juarez-Barber, Marina Segura-Benítez, Amparo Faus, Alexandra Trelis, Javier Monleón, Greta Carmona-Antoñanzas, Antonio Pellicer, James M. Flanagan, Hortensia Ferrero

**Affiliations:** 1 Fundación IVI, Instituto de Investigación Sanitaria La Fe, 46026 Valencia, Spain; 2 Departamento de Pediatría, Obstetricia y Ginecología, Universidad de Valencia, Valencia, Spain; 3 Department of Surgery and Cancer, Imperial College London, London, UK; 4 Hospital Universitario y Politécnico La Fe, Valencia, Spain; 5 Instituto de Medicina Genómica, Valencia, Spain; 6 IVIRMA Rome, Rome, Italy

**Keywords:** Histone modification, H3K4me3, Gene expression, Proliferation, Wnt/β-catenin, Uterine leiomyoma

## Abstract

**Background:**

Uterine leiomyomas (UL) are the most common benign tumor in women of reproductive age. Their pathology remains unclear, which hampers the development of safe and effective treatments. Raising evidence suggests epigenetics as a main mechanism involved in tumor development. Histone modification is a key component in the epigenetic regulation of gene expression. Specifically, the histone mark H3K4me3, which promotes gene expression, is altered in many tumors. In this study, we aimed to identify if the histone modification H3K4me3 regulates the expression of genes involved in uterine leiomyoma pathogenesis.

**Methods:**

Prospective study integrating RNA-seq (*n* = 48) and H3K4me3 CHIP-seq (*n* = 19) data of uterine leiomyomas versus their adjacent myometrium. Differentially expressed genes (FDR < 0.01, log2FC > 1 or < − 1) were selected following DESeq2, edgeR, and limma analysis. Their differential methylation and functional enrichment (FDR < 0.05) were respectively analyzed with limma and ShinyGO.

**Results:**

CHIP-seq data showed a global suppression of H3K4me3 in uterine leiomyomas versus their adjacent myometrial tissue (*p*-value< 2.2e-16). Integrating CHIP-seq and RNA-seq data highlighted that transcription of 696/922 uterine leiomyoma-related differentially expressed genes (DEG) (FDR < 0.01, log2FC > 1 or < − 1) was epigenetically mediated by H3K4me3. Further, 50 genes were differentially trimethylated (FDR < 0.05), including 33 hypertrimethylated/upregulated, and 17 hypotrimethylated/downregulated genes. Functional enrichment analysis of the latter showed dysregulation of neuron-related processes and synapsis-related cellular components in uterine leiomyomas, and a literature review study of these DEG found additional implications with tumorigenesis (i.e. aberrant proliferation, invasion, and dysregulation of Wnt/β-catenin, and TGF-β pathways). Finally, *SATB2, DCX, SHOX2, ST8SIA2, CAPN6,* and *NPTX2* proto-oncogenes were identified among the hypertrimethylated/upregulated DEG, while *KRT19, ABCA8,* and *HOXB4* tumor suppressor genes were identified among hypotrimethylated/downregulated DEG.

**Conclusions:**

H3K4me3 instabilities alter the expression of oncogenes and tumor suppressor genes, inducing aberrant proliferation, and dysregulated Wnt/β-catenin, and TGF-β pathways, that ultimately promote uterine leiomyoma progression. The reversal of these histone modifications may be a promising new therapeutic alternative for uterine leiomyoma patients.

**Supplementary Information:**

The online version contains supplementary material available at 10.1186/s12958-023-01060-2.

## Background

Uterine leiomyomas (ULs), or uterine fibroids, are benign tumors arising from smooth muscle cells located in the myometrium (MM). This highly prevalent gynecological disorder affects up to 70% of Caucasian women and more than 80% of women of African descent [1, 2]. However, only 30% of affected women present symptoms such as excessive uterine bleeding, dyspareunia, dysmenorrhea, infertility, and poor obstetrical outcomes [2]. Although the development of ULs has been associated with several dysregulated mechanisms involving genetic mutations [3], dysregulation of steroid hormones [4], Wnt/β-catenin [5], and TGF-β signaling pathways [6], the pathogenesis of this condition remains unclear. Nonetheless, other risk factors such as race, diet, age, body mass index (BMI), and parity [7] suggest the potential involvement of epigenetics in UL development.

The gold standard treatments for patients who suffer from this disorder are hysterectomy or myomectomy, despite the economic and personal consequences these surgeries impose [2]. Less invasive hormonal treatments are also currently available, however, they may cause menopausal side effects or hepatic damage [8] and are not as efficient [i.e., when treatment is discontinued the leiomyomas enlarge again [9]]. For these reasons, there exists a need for an alternative and effective UL treatment with minimal side effects. Broadening the knowledge of UL pathophysiology could lead to the identification of new molecular mechanisms involved in its pathogenesis, and consequently, the development of new treatments.

Epigenetics is defined as the variations in gene expression that are not caused by changes in the DNA sequence. The main epigenetic processes are DNA methylation, histone modification, and regulation by non-coding RNAs [10]. These modifications are inherited somaticlly and are reversible, thus becoming potential therapeutic targets. Specifically, histone modifications occur at the N-terminal tail of the globular domains of the core histones [11] and include acetylation, methylation, phosphorylation, ubiquitination, and SUMOylation. Varying combinations of histone modifications comprise a histone code, that directs biological processes via the recruitment of specific chromatin-associated proteins, which lead to distinct gene expression patterns [12]. Methylation in histone 3 (H3) has been associated with the regulation of gene transcription. Unlike DNA methylation which has suppressive effects on gene expression, histone methylation has variable effects on gene expression depending on the specific histone protein and amino acid residue modified [13]. In particular, a single methylation mark of histone 3 at the 4th lysine (H3K4) is associated with the activation of gene expression [14], while trimethylation of the same loci (H3K4me3) promotes gene transcription [15, 16]. In fact, H3K4me3 has been found altered in many tumors (e.g., breast, colon, and cervical cancer), however, its role in UL pathogenesis requires further investigation. To date, another study has analyzed the histone modification profile and mRNA expression in UL compared to MM [17], but they did not evaluate the specific interaction of H3K4me3 with the gene expression. Thus, we aimed to describe whether the histone mark H3K4me3 regulates key pathways and driver genes involved in UL pathogenesis, through an in-depth integrative analysis of the transcriptome and H3K4me3 CHIP-seq profiles in ULs compared to their adjacent MM, regardless their mutational subtype. With this study, we describe for the first time the functional implications of differential H3K4me3 promotor status on gene expression of DEGs in UL compared to its adjacent MM.

## Methods

### Data collection

Datasets from RNA-seq (GSE192354 and GSE142332) and H3K4me3 CHIP-seq (GSE142332) analyses of ULs and their adjacent MM were downloaded from the GEO database (https://www.ncbi.nlm.nih.gov/geo/). Of note, GSE192354 contained transcriptomic data of 31 matched tissues taken from Caucasian premenopausal women (aged 31–48) who had not received any hormonal treatment for the previous 3 months and who were undergoing myomectomy or hysterectomy due to symptomatic UL. GSE142332 contained RNA-seq and CHIP-seq H3K4me3 data of matched UL and MM tissues from 21 patients (aged 41–52) with diverse ethnicity (comprising 6 African-Americans, 9 Caucasian, and 6 with unknown status). 10 of these 21 patients were on hormonal treatment before surgery.

### Analysis of H3K4me3 CHIP-seq data

For the analysis of the histone mark H3K4me3 using CHIP-seq data from GSE142332, bioinformatic analysis was carried out using R/Bioconductor (version 4.1.1.). The biomaRt package was used to annotate genes from Ensembl to R. A matrix with the fold-enrichment for each signal value was created for those peaks up to 2 kb upstream/downstream from the transcription start sites (TSS) of a known human gene, which were considered target sites of histone modification. Fold-enrichment of signal values corresponding to peaks provided by CHIP-seq were normalized by standardization (value-median/standard deviation), and a Principal Component Analysis (PCA) and heatmap analysis were carried out. The boxplot of H3K4me3 histone mark status in UL and the MM was generated with the ggplot2 package. To assess the stability of the H3K4me3 status in ULs vs the MM, a Wilcoxon test was performed (*p* < 0.05 was considered significant). Of note, two samples (L19 and L21) were removed for insufficient quality, as demonstrated by their low sequence depth, as well as their corresponding myometrium.

### Identification of differentially expressed genes in uterine leiomyomas

The raw count matrix from the RNA-seq data libraries obtained from GSE192354 was processed and analyzed using R/Bioconductor (version 4.1.1.). A PCA was performed to analyze the concordance of the DNA libraries. Two libraries of GSE192354 (MM4 and MM20) resulted in very different PCA values (supplementary material, Fig. S1) and quality control metrics, and were not considered satisfactory and were therefore filtered out from downstream processing to avoid biased results. Differentially expressed genes (DEGs) were identified and analyzed using the DESeq2, edgeR, and limma packages, increasing the accuracy of the analysis. Following analysis of both RNA-seq data libraries, all DEGs with FDR-adjusted *p*-value< 0.01, log2FC > 1 or < − 1, and whose expressed was shared between both datasets, were considered relevant for UL pathogenesis and selected for further analysis.

### Assessment of H3K4me3 stability among differentially expressed genes

The selected DEGs were refined following integration with CHIP-seq data. Briefly, DEGs that had a CHIP-seq peak in the promoter region (TSS ± 2 kb) were chosen. A boxplot representing H3K4me3 status for downregulated and upregulated genes in UL and MM was created with the ggplot2 package. A Wilcoxon test was carried out to examine differences between H3K4me3 status in our samples (*p* < 0.05 was considered significant). Differential peak enrichment analysis was completed employing a linear model with the limma package for those normalized peaks that are present in the promoter region of the DEGs previously selected. Peaks within the regulator regions of the DEGs which had a FDR-adjusted *p*-value< 0.01 were considered to have a significantly unstable H3K4me3 status between ULs and the MM. Hypotrimethylated/downregulated and hypertrimethylated/upregulated were represented in a Venn diagram.

### Gene ontology enrichment analysis

Following the assessment of H3K4me3 stability, a Gene Ontology (GO) enrichment analysis was performed on the 50 DEGs which presented a differential status of H3K4me3 (17 hypomethylated/downregulated and 33 hypermethylated/upregulated genes) via Shiny Go (version 0.741) [18]. Biological processes and cellular components with FDR < 0.05 were considered to be epigenetically modified by H3K4me3.

### Validation cohort sample collection

For gene expression validation experiments, ULs and their adjacent MM matched tissues (*n* = 10) were obtained from a distinct cohort of Caucasian premenopausal women (aged 31–48 and BMI < 30). Two fragments of 2 cm^3^ each one corresponding to UL and MM were obtained during hysterectomy and smaller fragments of MM during myomectomy due to symptomatic UL indications at the Hospital Universitario y Politécnico (HUP) La Fe (Valencia, Spain). Adjacent MM was collected at a distance around 3 cm from the macroscopically observed tumor. The origin of these tissues was confirmed through haematoxylin/eosin staining by examination of a pathologist. This study was approved by the Clinical Ethics Committee of the HUP La Fe (ref. 2018/0097), and all participants provided written informed consent.

### qRT-PCR validation of gene expression

The gene expression of DEGs selected after the integration of RNA-seq and histone mark analysis was validated by quantitative real-time PCR (qRT-PCR) in a different set of samples to underline their importance in UL pathogenesis. TRIzol™ Reagent (Thermo Fisher Scientific, Waltham, MA, USA) was used to extract RNA from UL and MM samples, and complementary cDNA was synthesized using the PrimeScript RT reagent kit (Takara, Kusatsu, Japan). The expression of *CAPN6, NPTX2, SATB2, SHOX2, ST8SIA2, DCX, ABCA8, HOXB4,* and *KRT19* genes was analyzed by qRT-PCR, using the PowerUp™ SYBR™ Green Master Mix (Thermo Fisher Scientific) in a StepOnePlus™ system (Applied Biosystems, Waltham, MA, USA). These genes were selected from all hypermethylated/upregulated and hypomethylated/downregulated genes based on their roles in tumorigenesis and reported association(s) with the enriched functions. The Primer Quest Tool (Integrated DNA Technologies, Coralville, IA, USA) was used to design primers (Supplemental Table 1). Gene expression was normalized to GAPDH housekeeping gene expression, and fold change (FC) was calculated using the ΔΔCt method.

### Statistical analysis

Bioinformatics analyses were performed utilizing R (version 4.1.1.). Graphics were generated applying the R core package and packages plots, ggplot2, as well as GraphPad Prism 8.0. Validation analysis was conducted with GraphPad Prism 8.0, using the Student’s t-test or Wilcoxon test. *P* < 0.05 was considered statistically significant.

## Results

### H3K4me3 status in uterine leiomyomas compared to their adjacent myometrium

**Fig. 1:**
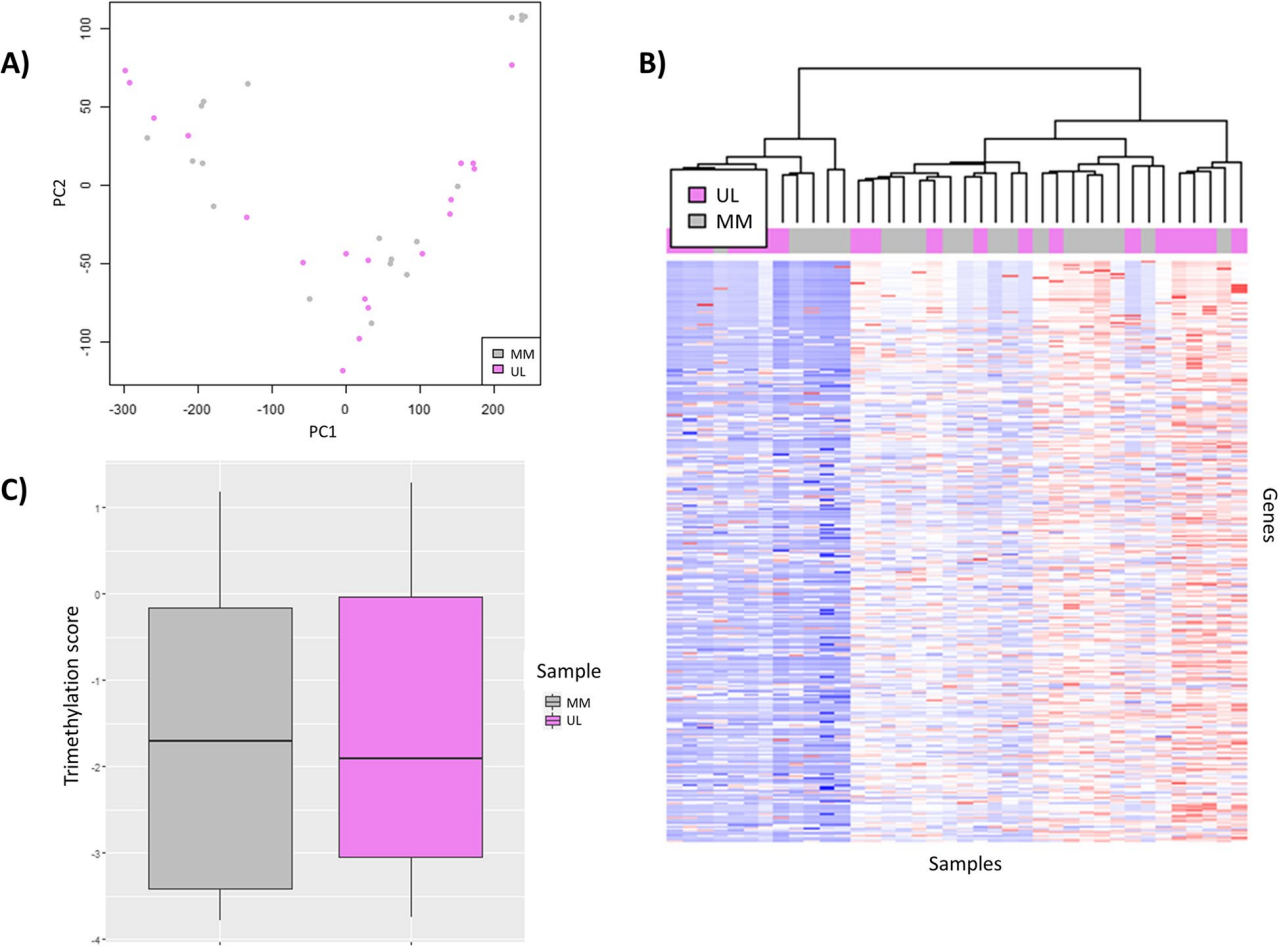
Overall H3K4me3 profile in uterine leiomyoma compared to adjacent myometrium tissues. Based on data extracted from GSE142332. **A** Principal component analysis of global H3K4me3 status in uterine leiomyomas (ULs; violet) and adjacent myometrium (MM; gray) (*n* = 19/group). **B** Heatmap representing the fold-enrichment score of genes with a CHIP-seq H3K4me3 peak in the promotor region after unsupervised clustering of ULs (violet) and MM (gray) (n = 19/group). The color scale ranges from red, for a higher normalized fold-enrichment score, to blue, for lower levels. **C** Boxplot analysis of the distribution of normalized fold-enrichment score for each peak in ULs (violet) compared to the adjacent MM (gray) samples (n = 19/group), representing the H3K4me3 profile

To describe the overall profile of the histone mark H3K4me3 in human UL compared to adjacent MM tissue, we performed an exploratory analysis of all peak signal values. In the preliminary PCA, we found similarities between ULs and their adjacent MM tissue (Fig. 1A). Clustering analysis also showed common patterns in UL and MM samples, which was corroborated by the heatmap analysis (Fig. 1B). Following the identification of genes that presented the H3K4me3 mark in their promotor region, the boxplot analysis showed a reduction in global H3K4me3 peak enrichment levels in ULs compared to MM tissue (*p*-value < 2.2e-16), indicating a global suppression of H3K4me3-mediated regulation of UL genes (Fig. 1C).

### Identification of significant differentially expressed genes in uterine leiomyoma tissue compared to adjacent myometrium

**Fig. 2:**
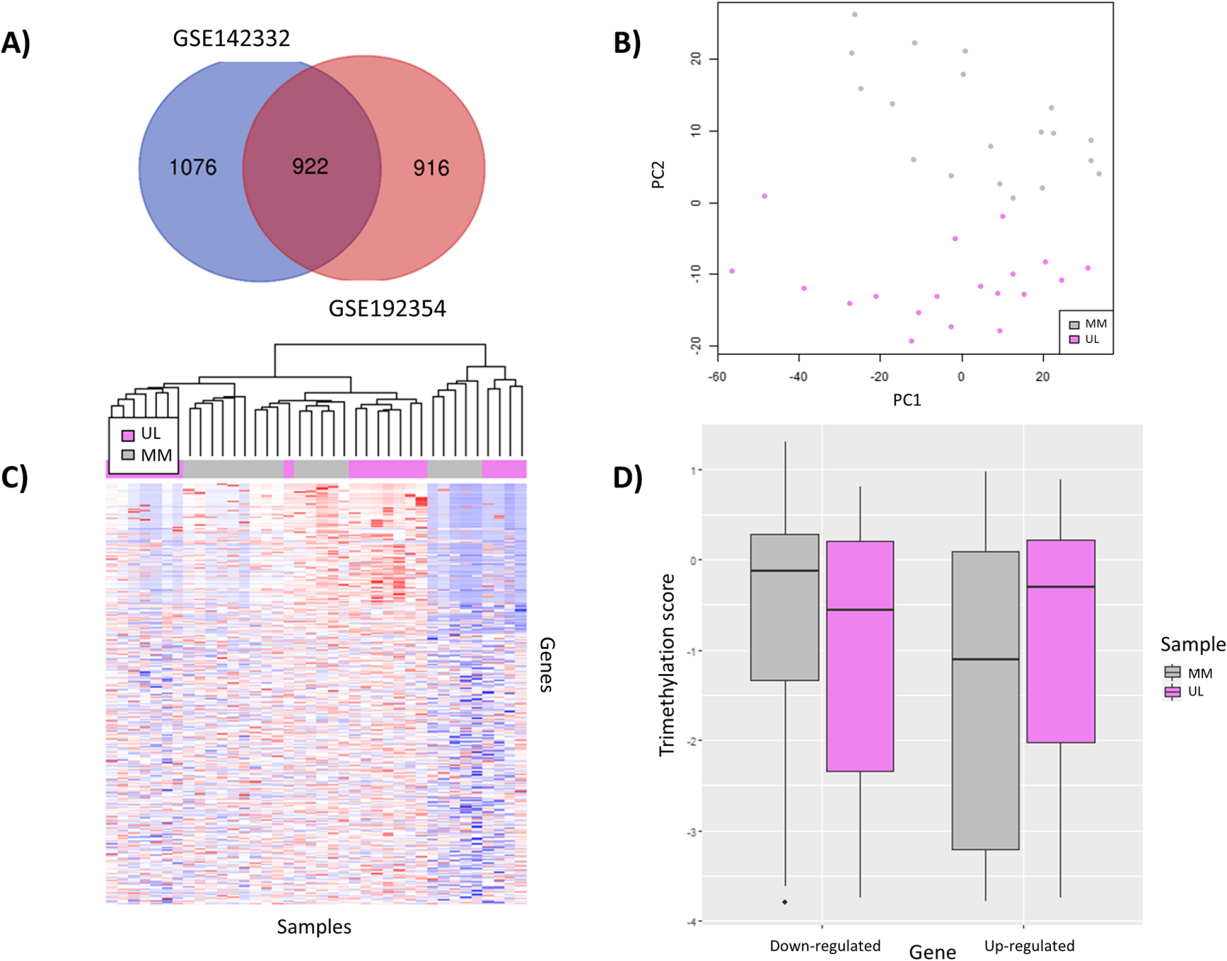
Selection of differentially expressed genes and description of their H3K4me3 status in ULs compared to MM tissue. **A** Venn diagram representing common DEGs (FDR-adjusted *p*-value< 0.01, log2FC > 1 or < − 1) between GSE192354 (*n* = 28) and GSE142332 (n = 19) datasets. **B** Principal component analysis of global H3K4me3 profile. **C** Heatmap based on the fold-enrichment score of selected DEGs shared between GSE192354 and GSE142332, whose promoter region presented a peak after unsupervised clustering of CHIP-seq data from uterine leiomyomas (ULs; violet) and their adjacent myometrium (MM; gray) (n = 19/group). The color scale ranges from red, for a higher normalized fold-enrichment score, to blue, for lower levels. **D** Boxplot analysis of the distribution of normalized fold-enrichment score for each peak of downregulated and upregulated genes in ULs (violet) compared to their adjacent MM (gray) samples (n = 19/group), representing the H3K4me3 status in each group of genes

To identify which DEGs could be implicated in UL pathogenesis, we integrated the transcriptomic data obtained by RNA-seq from two previous studies using independent patient cohorts [17, 19]. The gene expression count matrix from the GSE192354 dataset revealed 1837 significant DEGs (FDR-adjusted *p*-value< 0.01) with a substantial difference in expression between ULs and MM tissue (log2FC > 1 or < − 1), including 1175 upregulated and 662 downregulated genes. Likewise, 1998 significant DEGs (FDR-adjusted p-value< 0.01; log2FC > 1 or < − 1) were identified from the GSE142332 dataset, including 1106 upregulated and 892 downregulated genes. Integrating the DEGs obtained from both datasets unveiled 922 genes (of which 559 were upregulated and 363 downregulated) that were shared between ULs and the MM (Fig. 2A) and were selected for further analysis.

### Aberrant H3K4me3 mediates genomic instability in uterine leiomyomas

To assess the downstream impact of H3K4me3 in UL pathobiology, we analyzed the genes whose expression was altered in ULs concerning their adjacent MM due to H3K4me3 instabilities in the promoter region. Of the 922 DEGs selected following transcriptomic analysis, 696 presented the histone mark H3K4me3 around the promotor region. A PCA of the CHIP-seq data of these 696 DEGs showed a strong separation of UL and adjacent MM sample agruppations (Fig. 2B), indicating an unstable H3K4me3 profile in UL-relevant DEGs, which was corroborated by the heatmap (Fig. 2C). Subsequently, we analyzed the H3K4me3 status (hypermethylation/hypomethylation) of the 696 DEGs that presented the histone mark H3K4me3 based on whether they were downregulated or upregulated. The boxplot of the H3K4me3 signals revealed that upregulated genes exhibited significantly enhanced H3K4me3 signals in ULs versus the MM (Hypermethylation/upregulation; *p*-value < 2.2e-16), whereas downregulated genes presented significantly suppressed signals in ULs versus the MM (Hypomethylation/downregulation) p-value < 2.2e-16) (Fig. 2D). Finally, differential peak enrichment analysis indicated that, among these 696 DEGs, 50 DEGs presented differential trimethylation of H3K4 (FDR < 0.05) in ULs compared to the MM, including 33 hypertrimethylated/upregulated and 17 hypotrimethylated/downregulated regions (Supplemental Table 2).

### Functional implications of the aberrant H3K4 trimethylation in uterine leiomyomas

**Fig. 3:**
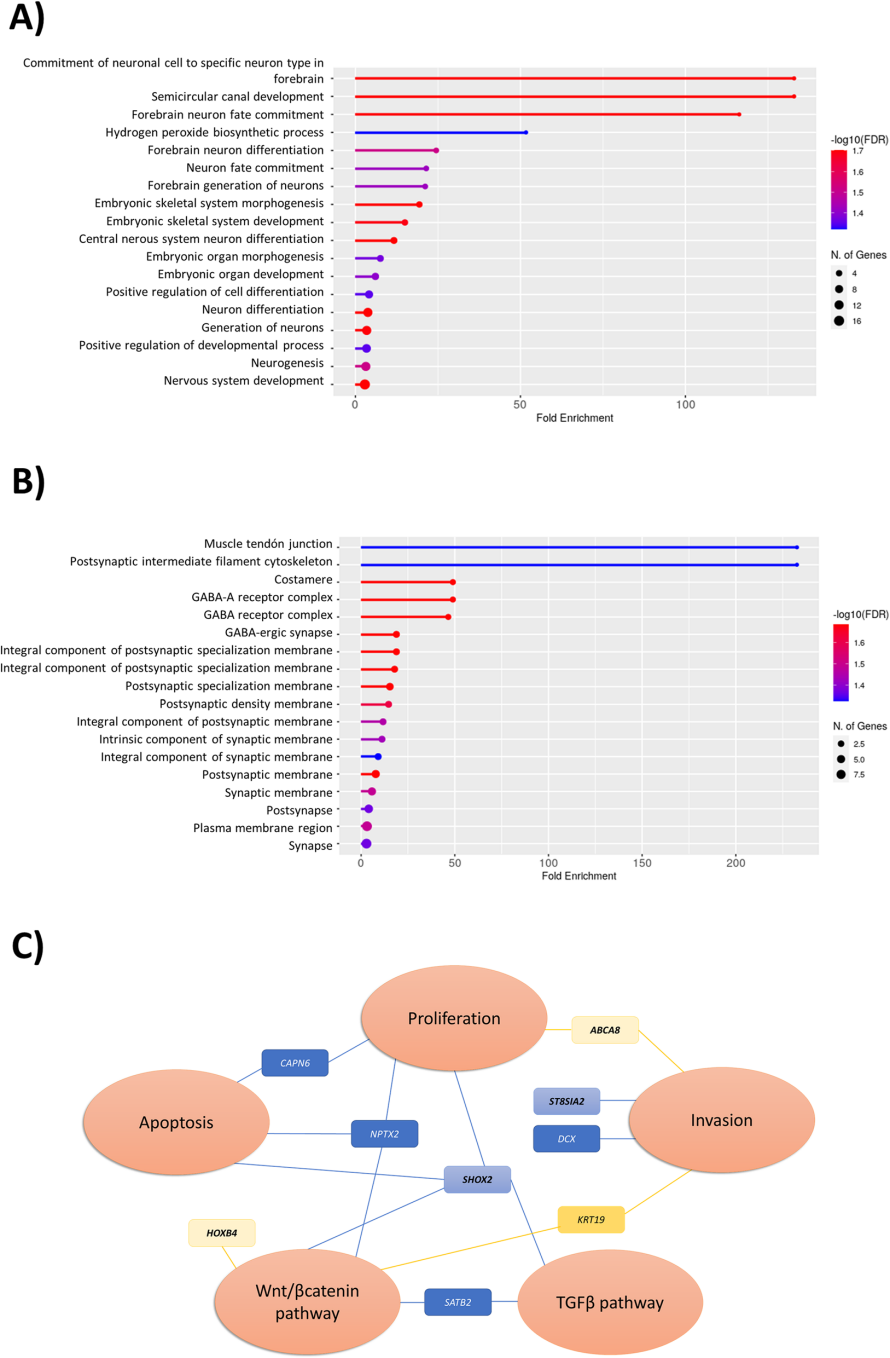
Functional enrichment of H3K4me3-mediated differential expressed genes with altered status in UL vs MM. The most significant (**A**) biological processes and (**B**) cellular components were obtained following functional enrichment analysis of common DEGs associated with H3K4me3 instabilities in UL vs MM tissues. **C** Functional implication of selected hypertrimethylated/upregulated oncogenes (blue squares) and hypotrimethylated downregulated genes (yellow squares). The genes that were not been previously related to UL are in bold

Gene ontology enrichment analysis of the 50 DEGs with fluctuating trimethylation of H3K4 in ULs revealed that neuron-related biological processes (Fig. 3A) and synapsis-related cellular components (Fig. 3B) were dysregulated. Beyond neuronal processes, these 50 genes have also previously been associated with tumorigenesis mechanisms such as aberrant proliferation, invasion, and dysregulation of Wnt/β-catenin and TGF-β pathways (Fig. 3C).

### Validation of hypotrimethylated/downregulated and hypertrimethylated/upregulated differentially expressed genes

**Fig. 4:**
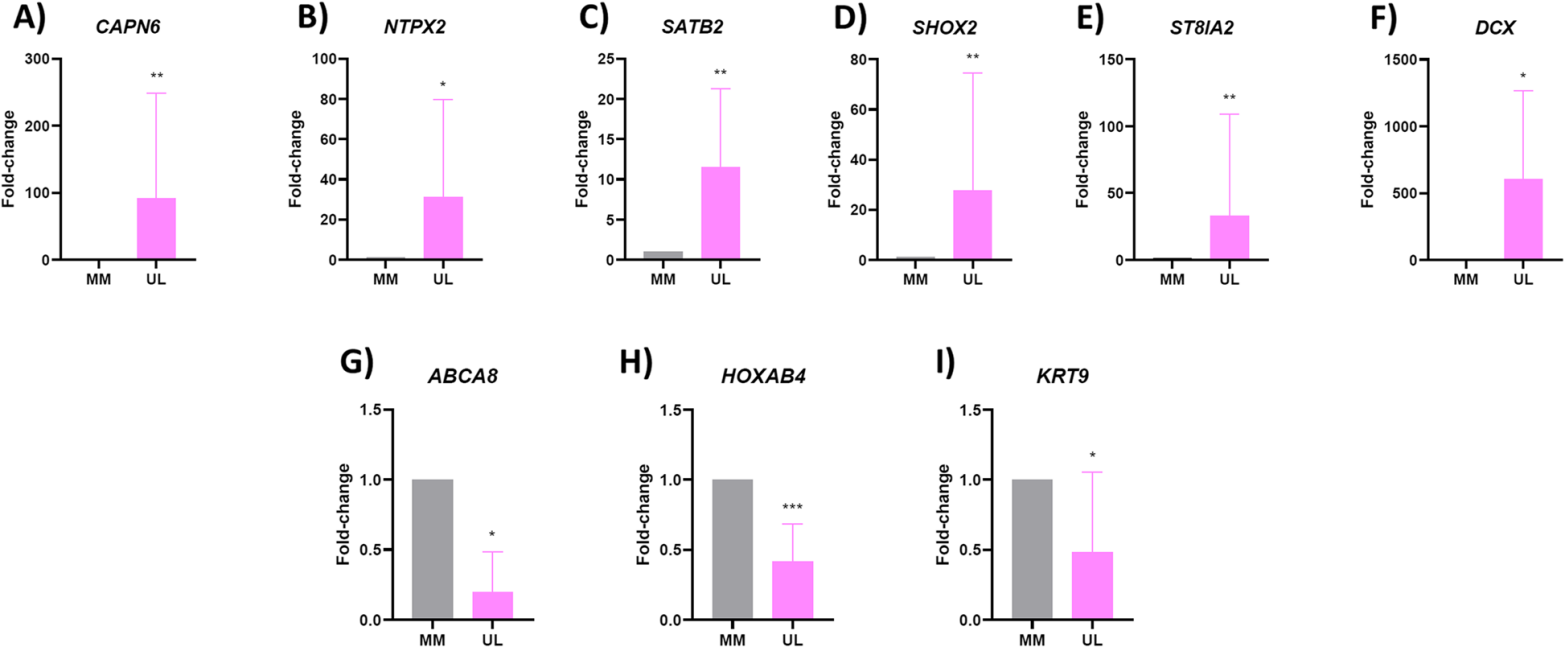
Validation of RNA-seq results. mRNA expression levels of (**A**) *CAPN6,* (**B**) *NPTX2,* (**C**) *SATB2,* (**D**) *SHOX2,* (**E**) *ST8SIA2,* (**F**) *DCX,* (**G**) *ABCA8,* (**H**) *HOXB4,* and (I) *KRT19* in a separate cohort of ULs compared to their adjacent MM matched tissues (*n* = 10). Relative gene expression was analyzed by qRT-PCR, quantified with the ΔΔCt method, and expressed as fold change. *p < 0.05; **p < 0.01; ***p < 0.001; ****p < 0.0001

To highlight the significance of the 50 genes selected with our in silico analyses, we selected nine genes with a differential H3K4me3, that we deemed most relevant for UL pathogenesis following a literature review [20–33]. Validation by qRT-PCR corroborated the significant upregulation of *CAPN6 (*FC = 91.79, *p* = 0.007), *NTPX2* (FC = 31.26, *p* = 0.015), *SATB2* (FC = 11.53, *p* = 0.005), *SHOX2* (FC = 27.62 p = 0.007), *ST8IA2* (FC = 33.17, *p* = 0.009), and *DCX* (FC = 602.56, *p* = 0.026) in a separate cohort of ULs compared to their adjacent MM (Fig. 4A–F). Likewise, qRT-PCR respectively confirmed the downregulation of *ABCA8* (FC = 0.19, p = 0.015), *HOXAB4* (FC = 0.41, *p* = 0.0004), and *KRT9* (FC = 0.29, *p* = 0.002) (Fig. 4G-I).

## Discussion

The role of epigenomic instability, via aberrant DNA methylation, has been recently considered for the onset and maintenance of ULs [19, 34–39]. Reversing the methylation could potentially restore gene expression and ultimately become a therapeutic alternative for women with ULs [19, 40]. The methylation in histones 3 (H3) and 4 (H4) is widely accepted to regulate gene transcription [13] and the profile of some histone modification has been analyzed in UL [17] The trimethylation of H3K4 is emerging as a key epigenetic modification in several cancers [14–16]. Specifically, the implications of histone methylation H3K4me3 on the expression of genes involved in UL development have never been evaluated in-depth. Thus, we studied for the first time the influence of H3K4me3 on UL-relevant genes to identify key pathways and driver genes involved in UL pathogenesis, and discovered novel targets for UL progression. Our results showed that H3K4me3 regulates the expression of oncogenes and tumor suppressor genes involved in neuronal processes and synaptic components, as well as key processes related to UL pathogenesis such as proliferation, dysregulation of the Wnt/β-catenin and TGF-β pathways).

In general, H3K4me3 activates gene expression. Using in silico analyses of the overall H3K4 trimethylation profile of gene promoter regions, we found significantly fewer H3K4me3 marks in ULs compared to their adjacent MM tissue, indicating a global hypotrimethylaion of H3K4 in UL and suggesting this epigenetic modification may be involved in the tumorigenic phenotype of ULs. To uncover the downstream effects of H3K4me3 on UL gene expression, we selected 922 relevant DEGs involved in UL development, following the integration of two independent RNA-seq datasets. Among these DEGs, 696 presented the histone mark H3K4me3 in their promoter region and 50 showed differential trimethylation of H3K4 in ULs compared to their adjacent MM tissue, including 33 hypertrimethylated/upregulated and 17 hypotrimethylated/downregulated.

The functional enrichment analysis of the 50 DEGs differentially affected by H3K4me3 revealed the most significantly enriched biological processes in UL were involved in the regulation of neuron-related processes, such as neurogenesis, neuron differentiation, and neuron fate commitment. In addition, the DEGs played active roles in cellular synapsis, involving cell components such as the postsynaptic intermediate filament cytoskeleton, GABA receptor complex, and synaptic membranes. These findings corroborate previous reports of the dysregulated expression of synaptic signaling and neuronal genes in the UL transcriptome [41, 42] and imply these genes are involved in UL-associated pain [42]. Furthermore, a recent in-depth review of the role of nerves in cancer not only highlighted their contribution to the tumor microenvironment but also described the reactivation of nerve-dependent developmental processes to promote cancer growth and survival [43].

Functional analysis of the hypertrimethylated/upregulated genes associated with UL pathogenesis revealed that most were oncogenes involved in tumor development. Among UL-associated genes, we observed H3K4me3-mediated regulation of *CAPN6, NPTX2, SATB2,* and *DCX.* The *CAPN6* protein, which regulates cell proliferation and apoptosis, is overexpressed in UL and plays an important role in tumorigenesis [20, 21, 26]. Meanwhile, *NPTX2* is a synaptic protein involved in malignancy via dysregulation of the cell cycle, apoptosis, and the Wnt/β-catenin pathway, in ovarian carcinoma [27], colorectal cancer [28], and UL [41]. Further, *SATB2* is hypermethylated and upregulated in most UL specimens, and activates the WNT/β-catenin and TGF-β signaling pathways which contribute to UL pathogenesis [29]. Finally, *DCX* is an oncogene implicated in the uncontrolled migration of cancer cells [30], which is upregulated in UL and leiomyosarcomas [21, 31]*.* Taken together, our findings suggest that the presence of the active histone mark H3K4me3 upregulates these genes in UL. Further, we identified for the first time two potential novel players in UL physiology, namely the hypertrimethylated/upregulated DEGs *SHOX2* and *ST8SIA2,* which are involved in tumorigenesis*.* The transcriptional regulator *SHOX2* plays a role in processing somatosensory information and is a known oncogene that regulates cell proliferation, apoptosis, WNT/β-catenin, and TGF-β signaling pathways in lung cancer [32]. On the other hand, *ST8SIA2* is an oncogene associated with aggressive disease and poor clinical prognosis in different cancers, including small cell lung cancer (SCLC), pancreatic cancer, and neuroblastoma [33]. The latter has been proposed as a druggable target, that has the potential to critically interfere with tumor cell dissemination in metastatic cancers [22, 33]. Thus, considering that UL is a benign tumor that share features with malignant tumours, we propose that *ST8SIA2* could be considered as a novel molecular target for UL treatment.

By examining the functions of hypotrimethylated/downregulated genes, we were able to shed light on the downstream effects of the epigenetic regulation of ULs. In this regard, we described the histone hypotrimethylation of *KRT19*, a tumor suppressor gene that has been previously described in UL [25]. The latter regulates cell migration, invasion, and metastasis through direct interaction with β-catenin [46], and is regulated by histone demethylase in prostate cancer cells [47]. Further, we revealed novel downregulated genes *ABCA8* and *HOXB4,* that had not previously been associated with UL. The *ABCA8* is a tumor suppressor gene whose downregulation transforms epithelium into mesenchyme, via the ERK/ZEB1 signaling pathway, promoting tumor progression [23]. Its low expression has been negatively correlated with prognosis in hepatocellular carcinoma [23], as well as ovarian cancer [24]. Alternatively, *HOXB4* is a transcription factor that is downregulated in cervical cancer cells and activates the Wnt/β-catenin signaling pathway [25].

It has been previously reported an association between H3K4me3 and H3K27ac domains [44]. For this reason, we analysed the DEGs with altered status of H3K4me3 and H3K27ac by comparing with our previous study [45]. We observed 13 DEGs with a differential status of H3K4me3 and H3K27ac, being 2 hypermethylated/hyperacetylated/upregulated and 11 hypomethylated/hypoacetylated/downregulated. Interestingly, *KRT19, ABCA8 and HOXB4* also present hypoacetylation of H3K27 on their promotor region. From the genes highlighted in our H3K27ac analysis, *DPT,* an hypoacetylated/downregulated is also hypomethylated, and regulates cell proliferation, TGF-β pathway and plays an important role in ECM [46]. Taken these studies together, we can suggest the importance of histones interaction in UL pathophysiology and its relevance for further studies.

Taken together, the dysregulated genes described in this study may be involved in the regulation of neuron-related processes and cellular synapsis, in addition to UL progression by altering proliferation, invasion, Wnt/β-catenin, and TGF-β signaling pathways. The Wnt/β-catenin pathway appears to be involved in UL emergence [47], and its regulators have been postulated as promising therapeutic targets, due to their aberrant activation in UL cells compared to MM cells [40, 48, 49]. On the other hand, the TGF-β signaling pathway is overexpressed in ULs compared to their adjacent MM tissue and is directly responsible for the development of the UL fibrotic phenotype through the increase of ECM deposition and cell proliferation [50–52]. Interestingly, treatment with the histone deacetylase inhibitor suberoylanilide hydroxamic acid (SAHA) inhibits the TGF-β signaling pathway and has proved to be a promising treatment for UL treatment [53]. Our findings open insights to the study of the relationship between H3K4me3 and Wnt/ β-catenin or TGFβ pathways and the regulation of these signaling pathways by this histone mark. Targeting H3K4me3 could alter both the Wnt/β-catenin and TGF-β pathway dysregulation in UL, as well as aberrant proliferation and invasion, providing an alternative therapeutic approach for UL treatment. This is a transcriptomic and epigenomic study that analyse the regulation of gene expression by H3K4me3 in which functional approaches were not performed. However, our findings pave the way to further functional studies, such as proteomic analysis, to assess the impact of this histone modification in UL pathogenesis and treatment.

## Conclusions

We revealed hypermethylation/upregulation of oncogenes such as *CAPN6, NPTX2, SATB2, SHOX2, ST8SIA2,* and *DCX*, in addition to hypomethylation/downregulation of tumor suppressor genes such as *ABCA8, HOXB4,* and *KRT1* in UL, that are not only involved in neuronal processes and with components of cell synapsis, but also elucidated underlying factors of UL pathogenesis, as aberrant proliferation, invasion, and Wnt/β-catenin and TGF-β pathways. These findings suggest that H3K4me3-mediated regulation of gene expression is involved in UL pathogenesis, and the reversion of this histone modification may be a promising therapeutic alternative for UL patients.

## Supplementary Information

**Additional file 1: Supplementary Figure 1.** Sample clustering of GSE192354. Principal component analysis of global transcriptome of the 60 samples obtained from GSE192354.

**Additional file 2: Supplemental Table 1.** qRT-PCR primer sequences of selected differentially expressed genes.

**Additional file 3: Supplemental Table 2.** Differentially expressed genes under H3K4me3 regulation in ULs compared to MM, with FDR-adjusted *p*-value< 0.01 after limma analysis.

## Data Availability

The GSE192354 and GSE142332 datasets analyzed herein are available in the GEO repository, https://www.ncbi.nlm.nih.gov/geo/.

## References

[1] Stewart EA, Laughlin-Tommaso SK, Catherino WH, Lalitkumar S, Gupta D, Vollenhoven B (2016). Uterine fibroids. Nat Rev Dis Prim.

[2] Giuliani E, As-Sanie S, Marsh EE (2020). Epidemiology and management of uterine fibroids. Int J Gynaecol Obstet.

[3] Mehine M, Kaasinen E, Heinonen HR, Mäkinen N, Kämpjärvi K, Sarvilinna N (2016). Integrated data analysis reveals uterine leiomyoma subtypes with distinct driver pathways and biomarkers. Proc Natl Acad Sci U S A.

[4] Maruo T, Ohara N, Wang J, Matsuo H (2004). Sex steroidal regulation of uterine leiomyoma growth and apoptosis. Hum Reprod Update.

[5] Ono M, Yin P, Navarro A, Moravek MB, Coon VJS, Druschitz SA (2013). Paracrine activation of WNT/β-catenin pathway in uterine leiomyoma stem cells promotes tumor growth. Proc Natl Acad Sci U S A.

[6] Ciebiera M, Włodarczyk M, Wrzosek M, Męczekalski B, Nowicka G, Łukaszuk K, et al. Role of transforming growth factor β in uterine fibroid biology. Int J Mol Sci. 2017;18(11):2435. 10.3390/ijms18112435 PMC5713402 29149020

[7] Stewart E, Cookson C, Gandolfo R, Schulze-Rath R (2017). Epidemiology of uterine fibroids: a systematic review. BJOG An Int J Obstet Gynaecol.

[8] Manyonda I, Sinthamoney E, Belli AM (2004). Controversies and challenges in the modern management of uterine fibroids. BJOG.

[9] Sohn GS, Cho S, Kim YM, Cho C-H, Kim M-R, Lee SR (2018). Current medical treatment of uterine fibroids. Obstet Gynecol Sci.

[10] Styer AK, Rueda BR (2016). The epidemiology and genetics of uterine leiomyoma. Best Pract Res Clin Obstet Gynaecol.

[11] Yang Q, Mas A, Diamond MP, Al-Hendy A (2016). The mechanism and function of epigenetics in uterine leiomyoma development. Reprod Sci.

[12] Bártová E, Kreǰcí J, Harničarová A, Galiová G, Kozubek S (2008). Histone modifications and nuclear architecture: a review. J Histochem Cytochem.

[13] Barski A, Cuddapah S, Cui K, Roh TY, Schones DE, Wang Z (2007). High-resolution profiling of histone methylations in the human genome. Cell.

[14] Hughes AL, Kelley JR, Klose RJ. Understanding the interplay between CpG island-associated gene promoters and H3K4 methylation. Biochim Biophys acta Gene Regul Mech. 2020;1863(8):194567. 10.1016/j.bbagrm.2020.194567 PMC7294231 32360393

[15] Berger L, Kolben T, Meister S, Kolben TM, Schmoeckel E, Mayr D (2020). Expression of H3K4me3 and H3K9ac in breast cancer. J Cancer Res Clin Oncol.

[16] Li S, Shen L, Chen KN (2018). Association between H3K4 methylation and cancer prognosis: a meta-analysis. Thorac Cancer.

[17] Leistico J, Saini P, Futtner C, Hejna M, Omura Y, Soni P, et al. Epigenomic tensor predicts disease subtypes and reveals constrained tumor evolution. Cell Rep. 2021;34(13):108927. 10.1016/j.celrep.2021.108927 PMC8111960 33789109

[18] Ge SX, Jung D, Yao R (2020). ShinyGO: a graphical gene-set enrichment tool for animals and plants. Bioinformatics.

[19] Carbajo-García MC, Corachán A, Juárez-Barber E, Monleón J, Payá V, Trelis A (2022). Integrative analysis of the DNA methylome and transcriptome in uterine leiomyoma shows altered regulation of genes involved in metabolism, proliferation, extracellular matrix, and vesicles. J Pathol.

[20] Chen L, Xiao D, Tang F, Gao H, Li X (2020). CAPN6 in disease: an emerging therapeutic target (review). Int J Mol Med.

[21] Xia L, Liu Y, Fu Y, Dongye S, Wang D (2017). Integrated analysis reveals candidate mRNA and their potential roles in uterine leiomyomas. J Obstet Gynaecol Res.

[22] Sato C, Kitajima K. Polysialylation and disease. Mol Asp Med. 2021;79:100892. 10.1016/j.mam.2020.100892 32863045

[23] Cui Y, Liang S, Zhang S, Zhang C, Zhao Y, Wu D, et al. ABCA8 is regulated by miR-374b-5p and inhibits proliferation and metastasis of hepatocellular carcinoma through the ERK/ZEB1 pathway. J Exp Clin Cancer Res. 2020;39(1):90. 10.1186/s13046-020-01591-1 PMC7236190 32430024

[24] Liu X, Gao Y, Zhao B, Li X, Lu YI, Zhang J (2015). Discovery of microarray-identified genes associated with ovarian cancer progression. Int J Oncol.

[25] Lei D, Yang WT, Zheng PS. HOXB4 inhibits the proliferation and tumorigenesis of cervical cancer cells by downregulating the activity of Wnt/β-catenin signaling pathway. Cell Death Dis. 2021;12(1):105. 10.1038/s41419-021-03411-6 PMC7820415 33479226

[26] Andrique C, Morardet L, Linares LK, Cissé MY, Merle C, Chibon F, et al. Calpain-6 controls the fate of sarcoma stem cells by promoting autophagy and preventing senescence. JCI insight. 2018;3(17):e121225. 10.1172/jci.insight.121225 PMC6171816 30185659

[27] Han X, Lu Y, Li X, Xia L, Wen H, Feng Z, et al. Overexpression of NPTX2 promotes malignant phenotype of epithelial ovarian carcinoma via IL6-JAK2/STAT3 signaling pathway under hypoxia. Front Oncol. 2021;11:643986. 10.3389/fonc.2021.643986 PMC7985451 33768003

[28] Xu C, Tian G, Jiang C, Xue H, Kuerbanjiang M, Sun L, et al. NPTX2 promotes colorectal cancer growth and liver metastasis by the activation of the canonical Wnt/β-catenin pathway via FZD6. Cell Death Dis. 2019;10(3):217. 10.1038/s41419-019-1467-7 PMC6399240 30833544

[29] Sato S, Maekawa R, Tamura I, Shirafuta Y, Shinagawa M, Asada H (2019). SATB2 and NGR1: potential upstream regulatory factors in uterine leiomyomas. J Assist Reprod Genet.

[30] Ayanlaja AA, Xiong Y, Gao Y, Ji G, Tang C, Abdullah Z, et al. Distinct features of Doublecortin as a marker of neuronal migration and its implications in Cancer cell mobility. Front Mol Neurosci. 2017;10:199. 10.3389/fnmol.2017.00199 PMC5487455 28701917

[31] Bae SM, Kim Y-W, Lee JM, Namkoong SE, Kim CK, Ahn WS (2004). Expression profiling of the cellular processes in uterine leiomyomas: omic approaches and IGF-2 association with leiomyosarcomas. Cancer Res Treat.

[32] Li N, Zeng Y, Huang J (2020). Signaling pathways and clinical application of RASSF1A and SHOX2 in lung cancer. J Cancer Res Clin Oncol.

[33] Al-Saraireh YMJ, Sutherland M, Springett BR, Freiberger F, Ribeiro Morais G, Loadman PM, et al. Pharmacological inhibition of polysialyltransferase ST8SiaII modulates tumour cell migration. PLoS One. 2013;8(8):e73366. 10.1371/journal.pone.0073366 PMC3739731 23951351

[34] Mlodawska OW, Saini P, Parker JB, Wei J-J, Bulun SE, Simon MA (2022). Epigenomic and enhancer dysregulation in uterine leiomyomas. Hum Reprod Update.

[35] George J, Fan H, Johnson B, Carpenter T, Foy K, Chatterjee A (2019). Integrated epigenome, exome, and transcriptome analyses reveal molecular subtypes and homeotic transformation in uterine fibroids. Cell Rep.

[36] Liu S, Yin P, Xu J, Dotts A, Kujawa S, Coon VJ, et al. Targeting DNA methylation depletes uterine leiomyoma stem cell-enriched population by stimulating their differentiation. Endocrinology. 2020;161(10):bqaa143. 10.1210/endocr/bqaa143 PMC7497820 32812024

[37] Yamagata Y, Maekawa R, Asada H, Taketani T, Tamura I, Tamura H (2009). Aberrant DNA methylation status in human uterine leiomyoma. Mol Hum Reprod.

[38] Maekawa R, Sato S, Yamagata Y, Asada H, Tamura I, Lee L, et al. Genome-wide DNA methylation analysis reveals a potential mechanism for the pathogenesis and development of uterine leiomyomas. PLoS One. 2013;8(6):e66632. 10.1371/journal.pone.0066632 PMC3688587 23818951

[39] Shen L, Song CX, He C, Zhang Y (2014). Mechanism and function of oxidative reversal of DNA and RNA methylation. Annu Rev Biochem.

[40] Carbajo-García MC, Corachán A, Segura-Benitez M, Monleón J, Escrig J, Faus A, et al. 5-aza-2′-deoxycitidine inhibits cell proliferation, extracellular matrix formation and Wnt/β-catenin pathway in human uterine leiomyomas. Reprod Biol Endocrinol. 2021;19(1):106. 10.1186/s12958-021-00790-5 PMC8265104 34233687

[41] Krsteski J, Gorenjak M, But I, Pakiž M, Potočnik U. Dysregulation of synaptic signaling genes is involved in biology of uterine leiomyoma. Genes (Basel). 2021;12(8):1179. 10.3390/genes12081179 PMC8394462 34440356

[42] Luddi A, Marrocco C, Governini L, Semplici B, Pavone V, Capaldo A (2019). Increased expression of neurogenic factors in uterine fibroids. Hum Reprod.

[43] Zahalka AH, Frenette PS (2020). Nerves in cancer. Nat Rev Cancer.

[44] Beacon TH, Delcuve GP, López C, Nardocci G, Kovalchuk I, van Wijnen AJ, et al. The dynamic broad epigenetic (H3K4me3, H3K27ac) domain as a mark of essential genes. Clin. Epigenetics. 2021;13(1):138. 10.1186/s13148-021-01126-1 PMC8264473 34238359

[45] Carbajo-garcía MC, de Miguel-Gómez L, Juárez-barber E, Trelis A, Monleón J, Pellicer A, et al. Deciphering the role of histone modifications in uterine leiomyoma: acetylation of H3K27 regulates the expression of genes involved in proliferation, cell signaling, cell transport. Angiogenesis Extracellular Matrix Formation Biomed. 2022;10(6):1279. 10.3390/biomedicines10061279 PMC9219820 35740301

[46] Arslan AA, Gold LI, Mittal K, Suen TC, Belitskaya-Levy I, Tang MS (2005). Gene expression studies provide clues to the pathogenesis of uterine leiomyoma: new evidence and a systematic review. Hum Reprod.

[47] El Sabeh M, Saha S, Afrin S, Islam M, Borahay M (2021). Wnt/β-catenin signaling pathway in uterine leiomyoma: role in tumor biology and targeting opportunities. Mol Cell Biochem.

[48] Corachán A, Ferrero H, Aguilar A, Garcia N, Monleon J, Faus A (2019). Inhibition of tumor cell proliferation in human uterine leiomyomas by vitamin D via Wnt/β-catenin pathway. Fertil Steril.

[49] Al-Hendy A, Diamond MP, Boyer TG, Halder SK (2016). Vitamin D3 inhibits Wnt/β-catenin and mTOR signaling pathways in human uterine fibroid cells. J Clin Endocrinol Metab.

[50] Lee B-S, Nowak RA (2001). Human leiomyoma smooth muscle cells show increased expression of transforming growth factor-β3 (TGFβ3) and altered responses to the Antiproliferative effects of TGFβ 1. J Clin Endocrinol Metab.

[51] Lewis TD, Malik M, Britten J, Parikh T, Cox J, Catherino WH (2019). Ulipristal acetate decreases active TGF-β3 and its canonical signaling in uterine leiomyoma via two novel mechanisms. Fertil Steril.

[52] Corachán A, Trejo MG, Carbajo-García MC, Monleón J, Escrig J, Faus A (2021). Vitamin D as an effective treatment in human uterine leiomyomas independent of mediator complex subunit 12 mutation. Fertil Steril.

[53] Carbajo-García MC, García-Alcázar Z, Corachán A, Monleón J, Trelis A, Faus A (2022). Histone deacetylase inhibition by suberoylanilide hydroxamic acid: a therapeutic approach to treat human uterine leiomyoma. Fertil Steril.

