# Supplementary figures and images for: H3K4me3 mediates uterine leiomyoma pathogenesis via neuronal processes, synapsis components, proliferation, and Wnt/β-catenin and TGF-β pathways
Source: Reprod Biol Endocrinol. 2023 Jan 26;21:9. doi: 10.1186/s12958-023-01060-2 (PMC9878797; doi:10.1186/s12958-023-01060-2)

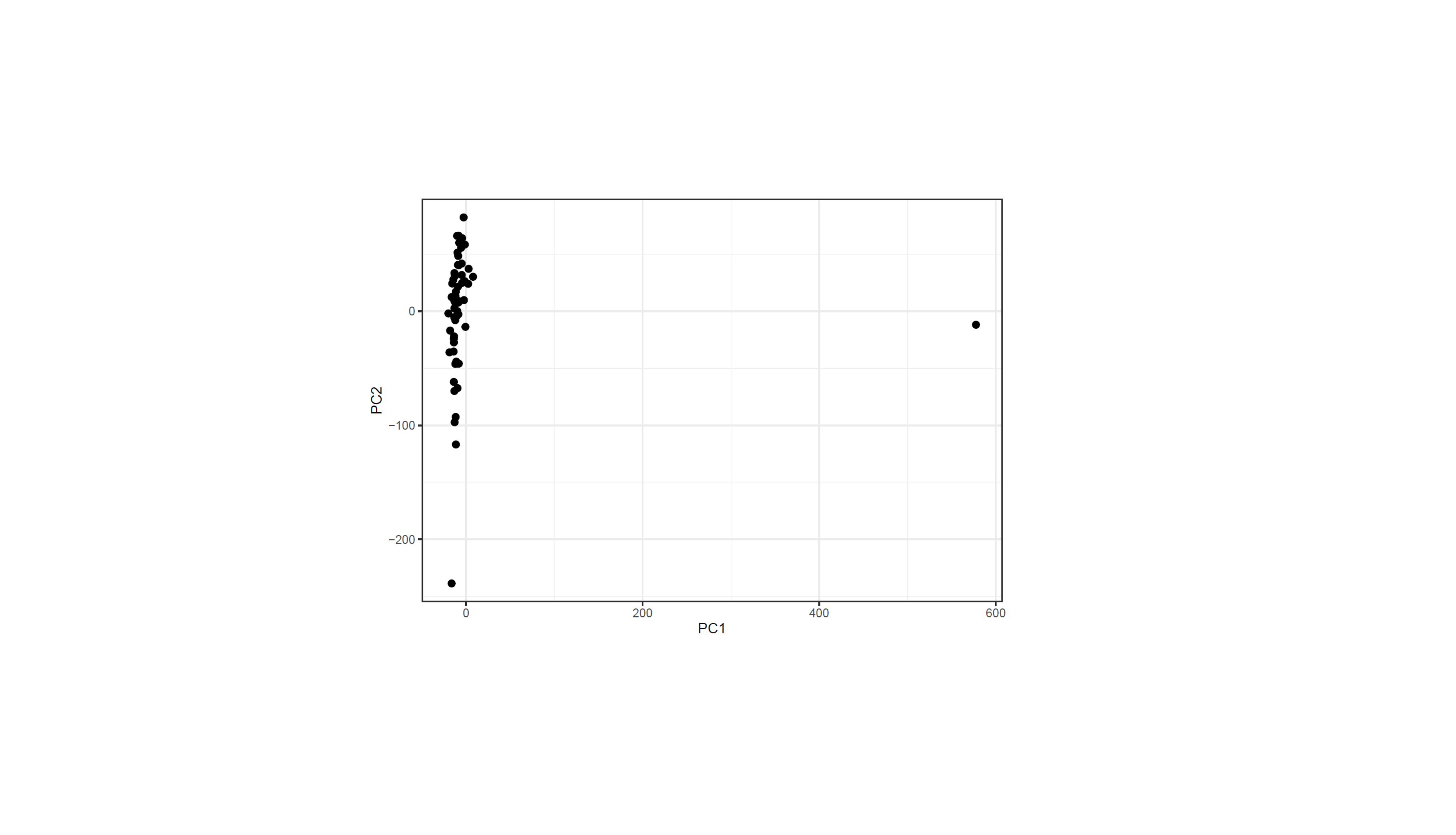

Supplement: Supplementary file 1 — Additional file 1: Supplementary Figure 1. Sample clustering of GSE192354. Principal component analysis of global transcriptome of the 60 samples obtained from GSE192354. [file 12958_2023_1060_MOESM1_ESM.tif]
