# Supplementary material for: H3K4me3 mediates uterine leiomyoma pathogenesis via neuronal processes, synapsis components, proliferation, and Wnt/β-catenin and TGF-β pathways
Source: Reprod Biol Endocrinol. 2023 Jan 26;21:9. doi: 10.1186/s12958-023-01060-2 (PMC9878797; doi:10.1186/s12958-023-01060-2)
Supplement: Supplementary file 2 — Additional file 2: Supplemental Table 1. qRT-PCR primer sequences of selected differentially expressed genes. [file 12958_2023_1060_MOESM2_ESM.docx]

**Supplemental Table 1.** qRT-PCR primer sequences of selected differentially expressed genes.

|  | GENE symbol | Forward sequence | Reverse sequence |
| --- | --- | --- | --- |
| *RT-qPCR* | *CAPN6* | GGTCTTCTCTTTCTCCACTTC | ATCCAGGGCCTCATAACA |
|  | *NPTX2* | GCTCATCAACGACAAGGT | TTCTCTCCGTCCTGGAAT |
|  | *SATB2* | AGGAGTTTGGGAGATGGT | CTGGCCCAGAACACAATAG |
|  | *SHOX2* | CTTGTAGAGTCGCACCTTATG | CCTGAACCTGAAAGGACAAG |
|  | *ST8SIA2* | GTGGGTCAACGAGCTTATC | TTTGTTGGTCAGCCAGTATC |
|  | *DCX* | CATGGATGAACTGGAGGAAG | GACAGACCAGTTGGGATTG |
|  | *ABCA8* | CGGGCTCTTCTTTGTTTCT | CATCAGGGATGTGCTGTTTA |
|  | *HOXB4* | CTGGATGCGCAAAGTTCA | TTCCTTCTCCAGCTCCAA |
|  | *KRT19* | TGGCCTACCTGAAGAAGAA | AATCCACCTCCACACTGA |
|  | *GAPDH* | AGATCAAGAAGGTGGTGAAG | TTFTCATACCAGGAAATGAGC |
